# Supplementary material for: Stacking Model‐Based Classifiers for Dealing With Multiple Sets of Noisy Labels
Source: Biom J. 2025 Mar 12;67(2):e70042. doi: 10.1002/bimj.70042 (PMC11898607; doi:10.1002/bimj.70042)
Supplement: Supplementary file 1 — Supporting Information [file BIMJ-67-e70042-s001.zip › density-based-ensemble-model-main/competitors_real_data/netlab-master/netlab-master/help/hbayes.htm]

Netlab Reference Manual hbayes


# hbayes

## Purpose

Evaluate Hessian of Bayesian error function for network.

## Synopsis

```
h = hbayes(net, hdata)
[h, hdata] = hbayes(net, hdata)
```

## Description

`h = hbayes(net, hdata)` takes a network data structure `net` together
the data contribution to the Hessian
for a set of inputs and targets.
It returns the regularised Hessian using any zero mean Gaussian priors
on the weights defined in
`net`. In addition, if a `mask` is defined in `net`, then
the entries in `h` that correspond to weights with a 0 in the
mask are removed.

`[h, hdata] = hbayes(net, hdata)` additionally returns the
data component of the Hessian.

## See Also

`gbayes`, `glmhess`, `mlphess`, `rbfhess`

---

**Pages:**
Index


---

Copyright (c) Ian T Nabney (1996-9)
